# Superoxide-producing thermostable associate from the small intestines of control and alloxan-induced diabetic rats: quantitative and qualitative changes

**DOI:** 10.1186/s12902-022-01160-x

**Published:** 2022-10-18

**Authors:** R. M. Simonyan, K. V. Simonyan, G. M. Simonyan, H. S. Khachatryan, M. A. Babayan, M. H. Danielyan, L. V. Darbinyan, M. A. Simonyan

**Affiliations:** 1 H. Buniatyan Institute of Biochemistry NAS RA, 0014 Yerevan, Armenia; 2 Neuroendocrine Relationships Lab, Orbeli Institute of Physiology NAS RA, 0028 Yerevan, Armenia; 3 Histochemistry and Electron microscopy Lab, Orbeli Institute of Physiology NAS RA, 0028 Yerevan, Armenia; 4 Sensorimotor Integration Lab, Orbeli Institute of Physiology NAS RA, 0028 Yerevan, Armenia

**Keywords:** Superoxide, Associate, Small intestine, Alloxan diabetes

## Abstract

**Background:**

NADPH oxidase 1 (Nox1), which is highly expressed in the colon, is thought to play a potential role in host defense as a physical and innate immune barrier against commensal or pathogenic microbes in the gastrointestinal epithelium. Diabetes can be caused by several biological factors, including insulin resistance is one of them. Alloxan is widely used to induce insulin-dependent diabetes in experimental animals. Alloxan increases the generation of reactive oxygen species as a result of metabolic reactions in the body, along with a massive increase in cytosolic calcium concentration.

**Methods:**

Using a universal method, a superoxide radical (О_2_^−^)-thermostable associate between NADPH-containing lipoprotein (NLP) and NADPH oxidase (Nox)- NLP-Nox was isolated and purified from the small intestine (SI) of control (C) and alloxan-induced diabetic (AD) albino rats.

**Results:**

In comparison to the C indices, in AD in the SI, an increase in the specific content of NLP-Nox associate and a decrease in the stationary concentration of produced О_2_^−^ in liquid phase (in solution) and gas phase (during blowing by oxygen of the NLP-Nox solution) were observed. The NLP-Nox of SI associate in C and AD rats produced О_2_^−^ by an immediate mechanism, using NLP as a substrate. The phenomenon of the hiding of the optical absorption maxima of the Nox in oxidized states at pH10,5 was observed in the composition of these SI associates of the C and AD rat groups. The characteristic absorption maxima of the «hidden» Nox were observed under these conditions after reduction by potassium dithionite.

**Conclusion:**

Thus, at AD, the decrease in the stationary concentration of produced О_2_^−^ in the solution and gas phase was compensated for by an increase in the specific amount of associate. In addition,  the decrease in the stationary concentration of produced О_2_^−^ by NLP-Nox associates at AD can be linked to a decrease in the level of NADPH in NLP-Nox composition. This could be used as a new mechanism of AD pathogenesis.

## Background

Insulin resistance and deficiency are associated with hyperglycemia and hyperlipidemia, leading to the development of many chronic diseases, such as neuropathy, nephropathy, and various vascular diseases related to the heart, kidneys, brain, peripheral vessels, and retinopathy. Diabetes can be caused by a number of biological factors including obesity, stress, excessive alcohol consumption, and improper exercise [1, 2].

Alloxan is an organic compound, urea derivative, and cytotoxic glucose analog. Alloxan is widely used to induce insulin-dependent diabetes (type 1) in experimental animals. It acts by increasing the generation of reactive oxygen species as a result of metabolic reactions in the body, along with a massive increase in cytosolic calcium concentration, and can rapidly cause pancreatic beta cell destruction [3–6].

NADPH oxidase 1 (Nox1), which is highly expressed in the colon, is thought to play a potential role in protecting the body as a physical and innate immune barrier against commensal or pathogenic microbes in the gastrointestinal epithelium. Vitamin E has been shown to improve SI changes in diabetic rats. These effects may be partially mediated by an increase in plasma antioxidant capacity and a decrease in lipid peroxidation [8–10]. It is well known that intravital NADPH fluorescence lifetime imaging in the SI of fluorescent reporter mice can be used to monitor NADPH-dependent metabolism of epithelial and myeloid cells. Diabetes causes numerous morphological and functional changes in the small intestine. After 6 weeks of diabetes, there was a fourfold increase in lipid peroxidation, as measured by thiobarbituric acid reactive substances, and a 38% increase in protein oxidation, as measured by protein carbonyl content. There was a significant increase in the activities of catalase (123.9%) and superoxide dismutase (71.9%) and a decrease in the activity of glutathione peroxidase (67.7%) [11–13]. We have shown previously that natural non-discontinuous permanent and stable О_2_^−^-producing system in the spinal cord injured rats with experimental type II diabetes are significantly destabilized. This destabilization was considerably intensified in diabetic rats subjected to additional spinal cord injury. Correspondingly, improvement of the antioxidant status may be an important component in the strategy of treatment of both diabetes and consequences of spinal cord injury [14]. Thus, these results suggest that oxidative stress occurs in the SI during diabetes and may be involved in some of the associated functional changes. The gastrointestinal epithelium serves as a physical and innate immune barrier against commensal and pathogenic microbes. NADPH oxidase 1 (Nox1) and dual oxidase 2 (Duox2), which are highly expressed in the colon, may play a role in the host defense. ROS derived from Nox1 have been linked to the pathogenesis of inflammation-related tumor development. The human stomach does not express Nox1 [10, 11,15]. The thermostable associate from the SI of C and alloxan diabetic rats can be obtained using a universal method [7] for preparing isoforms of thermostable associates from biomembranes of animal origin.

The goal of this study was to isolate and purify NLP-Nox isoforms from the SI of C and AD rats by determining the specific content of associates and the stationary concentration of produced О_2_^−^ in the solution and gas phase.

## Method

Diabetes was induced in rats by intraperitoneal administration of alloxan. Rats were divided into two groups (eight rats each): the control group (C) and the alloxan-induced diabetic group. Prior to the experiments, rats (220–240 g) were fed standard rodent food for one week for acclimation to the laboratory conditions. The acclimated rats were fasted for 12 h with free access to water, and then injected with 0.5% alloxan dissolved in 10 mM sodium citrate (pH 4.5) at a dose of 150 mg/kg. Under the same conditions, C rats were injected with a saline solution. After 5 days, blood glucose was measured using a «Saterlit» glucometer. After 5 days, the animals were fasted, anesthetized with halothane, and dissected [16].

The experimental protocol corresponded to the conditions of the European Communities Council Directive (2010/63/ UE) and was approved by the Ethics Committee of Yerevan State Medical University after Mkhitar Heratsi (IRB Approval N4, November 15, 2018).

### Isolation and purification of О_2_^−^-producing associate NLP- Nox from rat`s SI

NLP – Nox associates from rat SI were isolated and purified using a universal method [7], which uses human ferrihemoglobin (Hb) for the release of Nox and NLP-Nox from SI [17]. In particular, a water homogenate of SI (up to 6 g) was incubated for 1.5 h at pH 9,5 and 37 °C in the presence of 50 mM human Hb. The pH of the supernatant was adjusted to 4 after centrifugation at 5000 g for 10 min. The precipitate of the NLP-Nox fraction was soluble in water at pH9.5. After centrifugation, the supernatant was subjected to ion-exchange chromatography using cellulose DE-52 at pH 9.5. The Nox-NLP associate was eluted free because it was not absorbed by the column. The Nox fraction was eluted with 0.2 M potassium phosphate buffer (PPB) at pH 7.4. Following the concentration of the Nox and NLP-Nox fractions, gel filtration was performed on a separate Sephadex G-100 column at pH 9.5. The fractions of NLP-Nox and Nox eluted with a symmetrical elution diagram were collected and vacuum lyophilized after deionization of the Nox and NLP-Nox associates. They were weighed and stored in closed containers under a nitrogen atmosphere at -10^o^C.

Electrophoresis of the Nox-NLP associates was performed on a 10% PAAG (polyacrylamide gel) for proteins of acidic or basic characteristics.

### Determination of NADPH in the composition of SI NLP-Nox

The presence of NADPH in the composition of SI NLP-Nox was determined using a spectrofluorimetric method that measured the fluorescence intensities in comparative units (F) at 430 nm with excitation at 370 nm [18].

### Determination of the lipid component in the SI NLP-Nox composition

The lipid component of SI NLP-Nox was determined by measuring the lipid peroxidation product, malondialdehyde (MDA) [19].

### Determination of the Nox in the NLP-Nox composition of SI

The Nox in the composition of NLP-Nox associate from SI was determined using the optical absorbance characteristic for Nox at 558, 525, and 418 nm in the reduced states by sodium dithionite.

### Determination of the stationary concentration of О_2_^−^produced by SI NLP-Nox associate

The stationary concentration of О_2_^−^ produced by NLP-Nox associate from SI was determined using the adrenaline method. The maximal optical absorbance of adrenochrome (at 500 nm), which is formed during the oxidation of adrenaline by produced О_2_^−^ [20], was determined. At the same, the stationary concentration (M) of produced О_2_^−^ is equal to the concentration of formed adrenochrome, with a molar extinction (E) of up to 750 M-1 cm-1. The stationary concentration (M) of О_2_^−^ produced by this associate NLP-Nox was determined in the homogeneous phase (in solution) and gas phase by determining the value of A500/E. The optical absorbance of adrenochrome, which is formed during the oxidation of adrenaline by air oxygen, was used as a control.

The content of the lipid component (malondialdehyde) in NLP-Nox associate from SI in AD rats was 22.4% higher than that in C rats.

The specific content of NLP-Nox was determined by weighing it after deionization and vacuum lyophilization and was expressed in mg per 1 g SI (mg/g).

The cellulose of DE-52 («Whatman», England), Sephadex G-100 («Pharmacia», Sweden), adrenaline («Sigma», USA), spectrophotometer «Cary 60» (USA), spectrofluorometer «Perkin-Elmer», (USA), centrifuge K-70D and K-24 «Janetzki» (Germany) were used during the investigation.

Statistical analysis of the results using the variational statistics method of Student-Fisher was performed to determine the validation criterion (M ± m).

## Results

These О_2_^−^-producing associates NLP-Nox from SI of C and AD rats did not undergo PAAG electrophoresis and remained aggregated on the gel tube entry. Indirectly, the purity of these associates is evidenced by the absence of strips of accompanying water-soluble proteins of acidic and basic nature during electrophoresis of these associates’ opalescent solutions on 10% PAAG tubes. The symmetry of the elution diagrams of the Nox-NLP associates after gel filtration through Sephadex G-100 and the unchanged optical spectral index (A280/A400) show the purity of these associates.

**Fig. 1:**
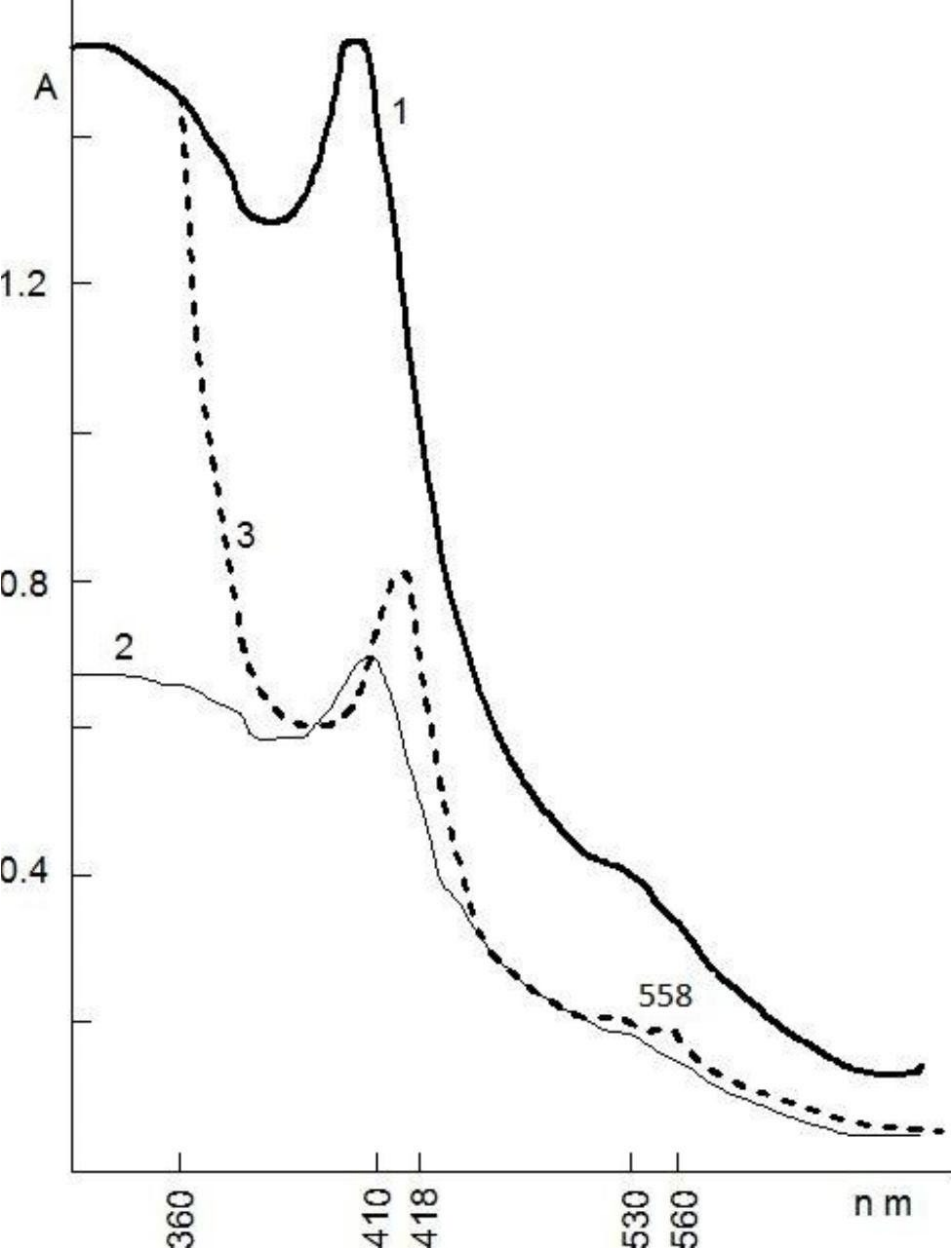
Optical absorption spectra of opalescent NLP-Nox associate solutions from SI of C (1) and AD rats (2) at pH 9.5. The spectra (3) were obtained after reduction of (2) by sodium dithionite

The specific content of NLP-Nox from SI of C and AD rats was 10,1 ± 0,12 mg/g and 16,6 ± 0,10 mg/g (p < 0,001, n = 6), correspondingly. After heating in boiling water for 10–12 min, the C and AD small intestine associates practically did not reduce nativity and О_2_^−^-producing activity. The characteristic absorption maxima for Nox were observed in the optical absorption spectra of NLP-Nox from SI, C, and AD rats in the oxidized and reduced states at pH 9,5 (Fig. 1).

**Fig. 2:**
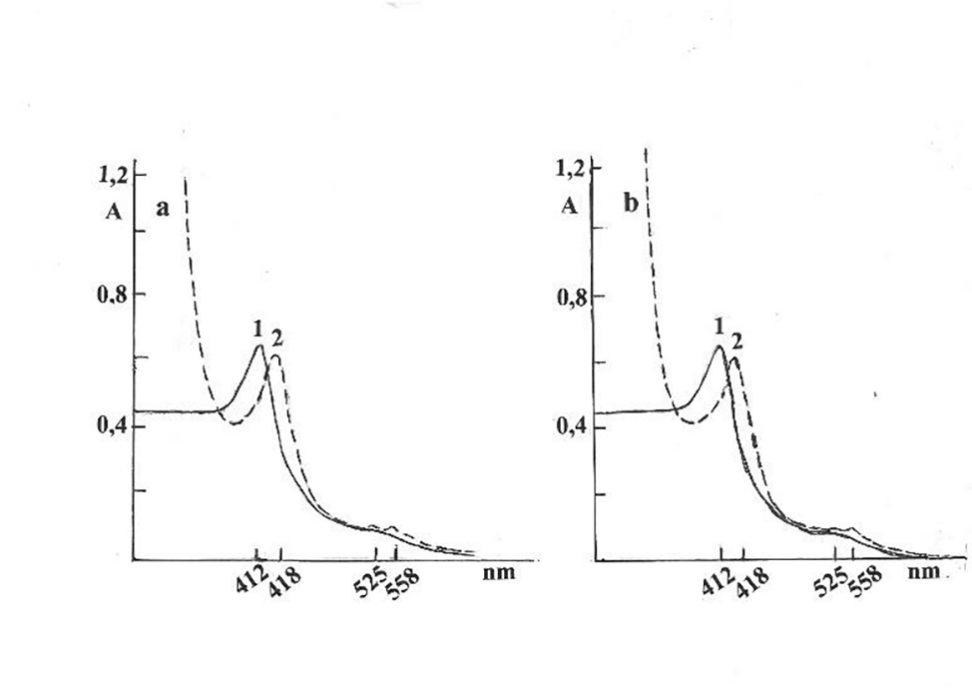
The optical absorption spectra of NOx from SI of C (a) and AD (b) rats in oxidized (1) and after reduction by sodium dithionite (2)

In comparison to Nox, in the associate of NLP-Nox the “free” Nox from SI in the associate of NLP-Nox had low oxidative/reductive properties, as shown in Fig. 2.

**Fig. 3:**
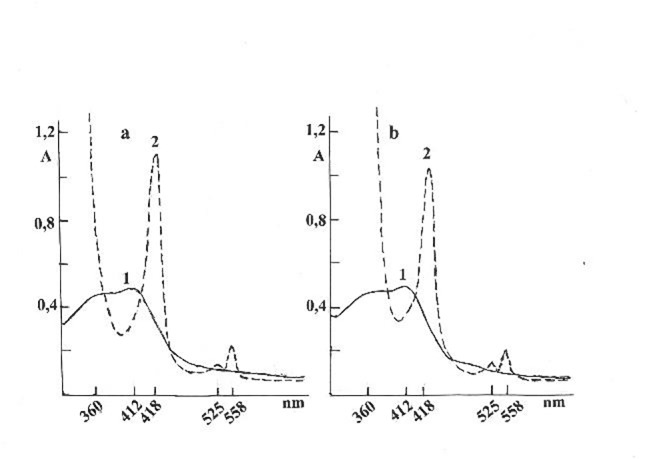
a – Optical absorption spectra of NLP-Nox associate from the SI of C rats in an oxidized state at pH 10,5 (1) and after reduction by sodium dithionite (2). b - Optical absorption spectra of the NLP-Nox associate from the SI of AD rats in an oxidized state (1) at pH10,5 and after reduction by sodium dithionite (2).

These results showed that the Nox in NLP-Nox associate was more easily reduced by sodium dithionite than free Nox from the SI of the C and AD rats. The slow opalescence of NLP-Nox associates from the SI of C and AD rats at pH 10,5 in oxidized and reduced states by sodium dithionite, which differed significantly, as shown in Fig. 3.

As shown in Fig. 3, the characteristic optical absorption spectra of Nox in the composition of the NLP-Nox associate did not exist in oxidized states at pH 10.5. However, the characteristic optical spectra of Nox in the composition of NLP-Nox were observed at pH 10.5 after the reduction of NLP-Nox associate with potassium dithionite. This phenomenon was observed for the first time in the present study. Reversible changes in the optical absorption spectra of NLP-Nox associates from the SI of the C and AD rats were observed.

**Fig. 4:**
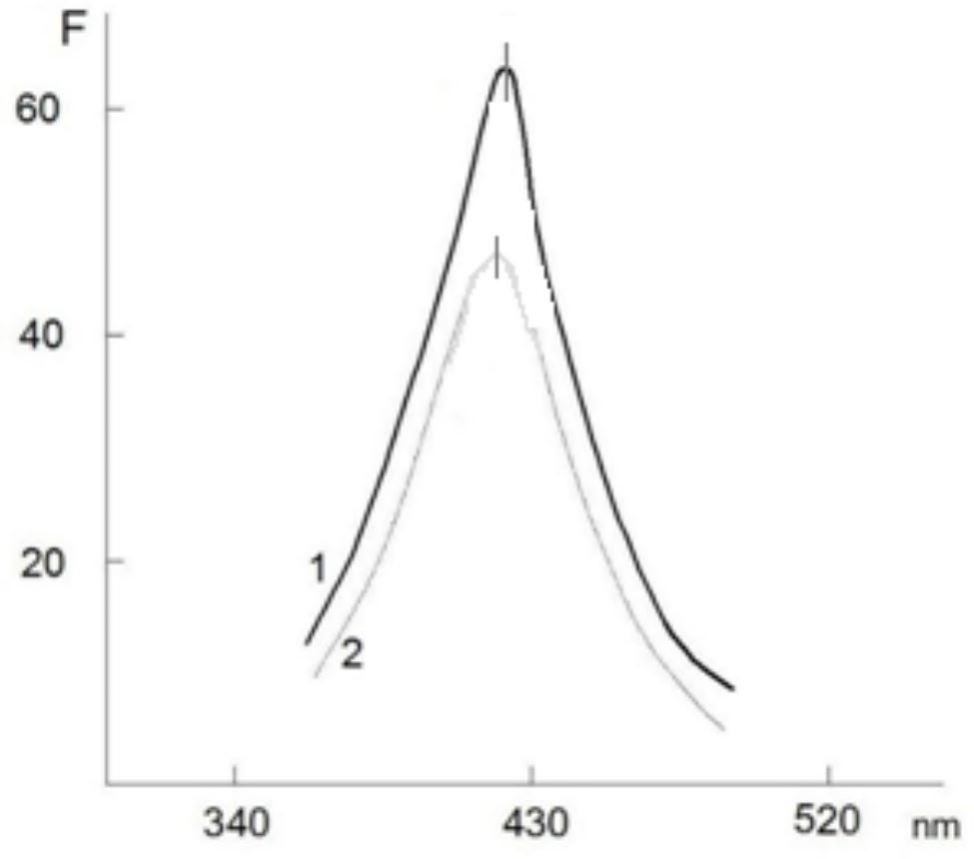
Spectrofluorescence spectra of NLP-Nox solution from the SI of C (1) and AD (2) rats (p < 0,005, n = 6)

The effect of NADPH on Nox activation in the gastrointestinal tract has already been revealed [11]. This NADPH, on the other hand, is a component of the NLP in the composition of the О_2_^−^-producing NLP-Nox associate from SI. In comparison to similar indicators in C rats, the fluorescence intensity «F» (in relative units) of NADPH in the composition of the associate of NLP-Nox from the SI of AD rats was observed up to 27,5 ± 1,6% (p < 0,005, n = 6) (Fig. 4).

The content of the lipid component (malondialdehyde) in NLP-Nox associate from the SI of AD rats is higher to 22,4%, than that in C rats.

The stationary concentrations of produced О_2_^−^ by NLP-Nox associates (1,4 mg/ml) in homogeneous phase (in solution) from C and AD rats were 5,6 ± 0,2 mM and 3,8 ± 0,2 mM (p < 0,05, n = 6).

## Discussion

Alloxan-induced diabetes animal models have been commonly used to study diabetes in vivo. Studies have shown that alloxan-diabetic rats are hyperglycemic due to damage to the insulin-secreting cells of the pancreas [21]. Many studies have indicated that alloxan alters the gut microbiota community in alloxan-diabetic rats [22]. For the first time, after blowing the NLP-Nox solutions with oxygen (0,2 atm, for 10 min at room temperature), the stationary concentration of produced and transferred into small silicone or glass tube gas phase О_2_^−^ by the NLP-Nox associate (1,4 mg/ml) from SI of C and AD rats is 4,8 ± 0,3 mM/ml (p < 0,001) and 3,2 ± 0,3 mM/ml (p < 0.001) were observed, correspondingly.

The decrease in the stationary concentration of produced О_2_^−^ by the NLP-Nox associate from SI of the AD rats can be linked to the decrease in the amount of NADPH in the composition of the NLP-Nox associate.

In fact, the oxygen is stabilized in the gas phase О_2_^−^ by forming a coordination band between О_2_ and О_2_^−^. On the other hand, it is known that the gas phase О_2_^−^ is formed: (a) in air by reducing molecular oxygen with traces of negative metal ions; (b) by an electrochemical method known as a “gas-phase superoxide generator”; (c) under the influence of earth crust radioactivity; and (d) during plant photosynthesis [23].

The enzymatically generated monocomponent gas phase О_2_^−^ from NLP-Nox associate from SI of the rats with regulated and effective stationary concentration of О_2_^−^ can be used by oxygen mask in experiments and, in the future, in clinics to treat lung infections. However, the monocomponent and regulated stationary concentration of enzymatically produced О_2_^−^ is preferred. NLP was used as a substrate in the composition of these associates.

Denaturation and decrease in superoxide production were not observed after boiling the associates in boiling water for 10–12 min. The higher thermostability of the О_2_^−^−producing NLP-Nox associates from SI of C and AD rats can be linked to the pulsed increase in temperatures up to 280-300^o^C during nanoseconds for the transmission of redox metabolic processes [24]. The following are the fundamental significance of the obtained results: (1) the immediate mechanism of the production of О_2_^−^ by the NLP-Nox associate from rats SI; (2) the formation of the monocomponent and regulated stationary concentration of gas phase О_2_^−^ form solutions of these NLP-Nox; and (3) the highly reductive properties of Nox in the composition of NLP-Nox associate from rats SI at pH 10.5. A family of NADPH oxidases is especially important for redox signaling and may be amenable to specific therapeutic targets. Nox isoforms are expressed in a cell- and tissue-specific manner, are subject to independent activation and regulation, and may have distinct functions. The complexity of regulation of NADPH oxidases may provide the possibility of targeted therapeutic manipulation in a tissue- and pathway-specific manner at appropriate points in the disease process [25]. The available data suggest that there is a perspective for the using of NCL from donor blood serum, as a potential Nox activator on the surface of immune cells at immunodeficiency in experiment (in which the О_2_^−^- producing activity of immune cells decreases [26]. Cells of immune system employ RNS/ROS to kill invading pathogens. A reduction of the redox state of immune cells may render immune cells less effective against invading organisms, resulting in an increased severity of the infection [27] and reduced form of NADPH for various cellular reactions including glutathione (GSH) recycling, superoxide anion production via NADPH oxidase, nitric oxide (NO) synthesis, and cholesterol synthesis [28], therefore quantitative and qualitative changes in NCL from blood serum can be used as new and sensitive diagnostic markers for various pathological conditions in experiments and clinics, in which a decrease in immune activity is observed.

The following are the implications of the obtained results:1) the qualitative and quantitative changes of these associates as new sensitive diagnostic tests in various pathological states and diseases, including gastrointestinal disorders and neurodegenerative diseases; 2) the use of gas phase О_2_^−^ as an antimicrobial and anticancer agent, stimulator of bone marrow stem cell proliferation, and so on.

## Conclusion

Our results suggest that the NLP-Nox associate from the rats’ small intestine is a new О_2_^−^-producing thermostable associate in the tissue. As a new AD diagnostic test, it immediately produces О_2_^−^ with the corresponding quantitative and qualitative changes in C and AD rats.

## Data Availability

All data generated and analyzed during this study are included in this article. The datasets used and/or analyzed during the current study are available from the corresponding author on reasonable request.

## Abbreviations

SI: small intestine
NLP: NADPH containing lipoprotein
C: control group
AD: alloxan-induced diabetes
Nox1: NADPH oxidase 1
MDA: malondialdehyde
